# Factors associated with women diagnosed with syphilis who received prenatal care in a primary healthcare unit

**DOI:** 10.31744/einstein_journal/2023AO0046

**Published:** 2023-03-07

**Authors:** Ana Lúcia de Lima Guedes, Daniela Cristina da Silva Guimarães, Diego Junqueira Sarkis, Tamiris Tiango Gabriel, Camila Silva Delgado, Angélica Atala Lombelo Campos, Mário Círio Nogueira, Luiz Cláudio Ribeiro

**Affiliations:** 1 Universidade Federal de Juiz de Fora Juiz de Fora MG Brazil Universidade Federal de Juiz de Fora , Juiz de Fora , MG , Brazil .

**Keywords:** Syphilis, congenital, Syphilis, Pregnancy complications, infections, Risk factors, Prenatal care, Primary health care

## Abstract

**Objective:**

To estimate the prevalence of syphilis and its associated factors in women who were treated at public maternity hospitals and received prenatal care in a primary healthcare unit.

**Methods:**

This cross-sectional study included 399 postpartum women. Interviews were conducted, and additional data were extracted from the pregnant woman’s booklet, medical records, and printed tests. The dependent variable was a gestational syphilis diagnosis. The independent variables were grouped into socioeconomic and demographic, behavioral, reproductive, and prenatal blocks. The prevalence, prevalence ratios, and 95% confidence intervals (95%CI) were calculated. The χ ^2^ test was also performed (p≤0.05). Multivariate analysis was performed using Poisson regression models.

**Results:**

The prevalence of gestational syphilis was 9.61% (95%CI: 7.14-12.83). We identified the following determining factors (adjusted prevalence ratios): history of sexually transmitted infections (2.3), first sexual intercourse by the age of 15 (2.42), partner having a history of syphilis (5.98), partner using crack/cocaine (6.42) and marijuana and others (3.02), not having a partner (3.07), low income (2.85), history of stillbirth (5.21), beginning prenatal care in the third trimester (3.15), and prenatal care received in a primary healthcare unit (without a Family Health Strategy team) (0.35).

**Conclusion:**

Individual and prenatal factors were associated with gestational syphilis. To control congenital syphilis, targeted interventions are needed to control syphilis in the adult population including expansion of access to quality prenatal care with identification of risks for syphilis and connection between prevention and treatment actions, implementation of strategies focused on early sexual education, effective establish prenatal care involving both partners, and effective implementation of the National Men’s Health Policy (PNAISH - *Política Nacional de Atenção Integral à Saúde dos Homens* ).

## INTRODUCTION

The World Health Organization (WHO) estimates that 2 million pregnant women develop syphilis annually, and approximately 25% of cases occur in the Americas. In 2015, there were 1.7 cases of congenital syphilis (CS) per thousand live births in Latin America and the Caribbean (LAC), corresponding to 85% of the estimated cases in Brazil. The number of cases of CS in Brazil almost doubled between 2010 and 2015. ^( 1 )^

The increase in acquired syphilis worldwide has contributed to an increase in gestational syphilis (GS) and CS. ^( 2 - 5 )^ Some risk factors include an increase in unprotected sex, ^( 3 - 5 )^ use of illicit drugs, ^( 4 , 5 )^ difficulty in accessing healthcare, ^( 1 - 3 , 5 , 6 )^ and a global shortage of penicillin. ^( 1 , 2 , 6 , 7 )^ In Brazil, the Ministry of Health has attributed three main factors to the increase in cases: increase in syphilis screenings, unavailability of penicillin, and lack of follow-up when patients are referred to secondary care. ^( 1 , 2 )^

The association between CS and inequalities in access to and quality of prenatal care has been well described. ^( 1 , 5 , 6 , 8 , 9 )^ The contexts of maternal vulnerability, such as poverty, low education, risky sexual behaviors, and other factors, can be directly associated with GS and CS, as they interfere with adequate prenatal care. ^( 5 , 7 - 10 )^

Prenatal care is one of the pillars of primary healthcare (PHC), which is the center of the regionalized and hierarchical model of healthcare and surveillance networks. ^( 11 , 12 )^ Primary health care follows the principles of the Brazilian Public Health System (SUS - *Sistema Único de Saúde* ) characterized by longitudinality, extramural action, proactive approach, and health responsibility of the teams, which defines a suitable profile for dealing with problems such as sexually transmitted infections (STIs) and CS. ^( 9 , 12 )^

Since 1995, Brazil has been a Pan American Health Organization signatory of the Plan to Eliminate CS as a public health problem, which is defined as an incidence rate ≤0.5 case of CS per thousand live births. The parameter was maintained and the documents renewed over the years; however, the elimination targets were not achieved. ^( 1 )^ Studies have revealed gaps in the prevention of GS and CS. Despite the low cost and effectiveness of early treatment of syphilis, preventive actions require interventions that include biomedical, behavioral, and sociocultural aspects. According to some authors, the care model adopted in Brazil in PHC units is adequate for the complexity of CS control; however, it did not incorporate the CS Elimination Project. ^( 9 , 12 )^ Knowledge of the vulnerabilities associated with GS can support effective measures aimed at improving access to and adequacy of prenatal care and preventive measures for CS.

## OBJECTIVE

To estimate the prevalence of syphilis and its associated factors in women treated at four public maternity hospitals for childbirth or abortion and who received prenatal care in a primary healthcare unit.

## METHODS

This study was conducted in Juiz de Fora, a large city in Minas Gerais. In the year of study (2018) there was an approximate coverage of *Atenção Básica* of 77% (Family Health Strategy [FHS] 57%). ^( 13 )^ The healthcare network offers treponemal and non-treponemal tests for the diagnosis of syphilis and penicillin for treatment, but there is no rapid testing or penicillin in the PHC units for immediate diagnosis and treatment. Approximately 80% of the births occurred in the four hospitals where the data were collected. The incidence rate (per 1000 live births) of CS in the municipality went from 0.6 in 2005 to 15.9 in 2018; this value is 30 times higher than the elimination target recommended by the WHO and the Ministry of Health. Between 2009 and 2018, 14.6% of the reported CS cases resulted in abortion or stillbirth. ^( 14 )^ This municipality has an incidence rate of CS that is almost twice as high as the averages for Minas Gerais and the nation. ^( 2 )^

A cross-sectional study was conducted from July to December 2018 and included 399 postpartum women hospitalized for childbirth or abortion. Those who received prenatal care at a PHC unit were eligible. Women with high risk complications, mental disorders, inability to establish verbal contact, and/or who did not carry their pregnant woman’s notebook at the time of data collection were excluded from the study.

The sample size was established considering the outcome “adequate prenatal care,” estimated at 50%, a significance level of 0.05, and corrected for finite population using an estimated population of 6,259 live births for mothers residing in the municipality in 2016. This number was increased by 10% due to possible losses, resulting in a sample of 399 women. Starting care in the first trimester and having had six or more appointments was considered “adequate prenatal care.”

The data were collected by the main researcher and four collaborators under supervision. The structured scripts and standardized questionnaires that were used were prepared by the research team, pre-tested, and evaluated in a pilot study. Interviews were conducted with the postpartum women, and additional data were extracted from pregnant women’s booklets, medical records, and printed tests brought by the pregnant women.

**Figure 1:**
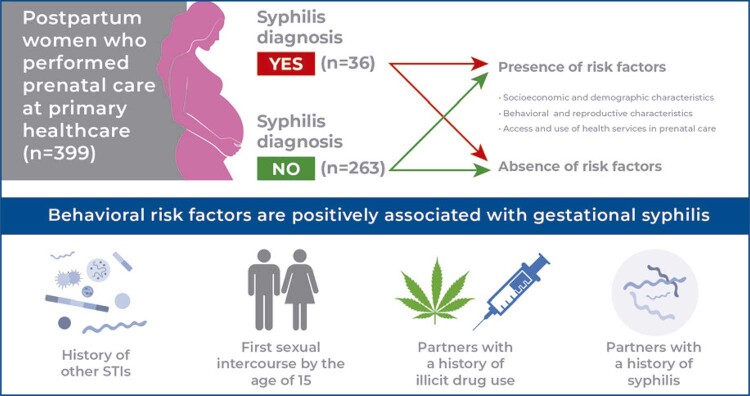
Flow diagram of the stages of identification, eligibility, and inclusion of participants

After coding and review, the database (Microsoft Excel, version 2013) was exported for analysis using STATA ^®^ (StataCorp, 2009; Stata Statistical Software: Release 11; StataCorp LP, College Station, Texas, USA). Figure 1 illustrates the flow diagram of the stages of identification, eligibility, and inclusion of participants. The outcome variable was “case of GS.” The independent variables were socioeconomic and demographic, reproductive, behavioral, and prenatal care factors.

Women with reactive syphilis tests in any titration (treponemal and/or non-treponemal) during pregnancy, childbirth, or postpartum were considered a “case of GS.” Seven (1.75%) women with reactive tests were not included as a “case of GS.” Five of them were considered “properly treated previous syphilis” (record of adequate previous treatment, no signs or symptoms, no risk of exposure, and a fall in VDRL titer post-treatment) and two were considered “false reagent results” (VDRL lower than the 1:4 dilution, no signs or symptoms, no risk of exposure, and non-reactive treponemal test).

The analysis of factors (independent variables) associated with GS was performed using a hierarchical model, with variables distributed into four blocks. The first, most distal level included socioeconomic and demographic characteristics about structural issues that have indirect effects on the outcome. Maternal characteristics (behavioral and reproductive) were included in the two intermediate levels and were considered to expand the understanding of more proximal determinants and their relationship with the outcome. Prenatal care characteristics were considered in the most proximal level. Variables on HIV testing were included in this block, as concomitant infections increase the potential for both sexual and vertical transmission of syphilis. ^( 1 , 2 )^

Qualitative variables are described using absolute and relative frequencies and quantitative variables using means and standard deviations. In the bivariate analysis, the proportions of independent variables were compared with the outcome. Prevalence ratios (PR) were estimated to determine the magnitude of the associations and accompanied by standard errors and 95% confidence intervals (95%CI). The χ ^2^ test was performed to detect differences between proportions (p≤0.05). Multivariate analysis was performed for each block and later between blocks using Poisson regression models. Variables with p≤0.20 in the bivariate analysis were included in the regression model in each block and those with p≤0.05 were retained. The final model included factors selected from each block, starting from the most distal to the most proximal to the GS outcome. Variables with p≤0.05 were retained.

This study is part of broader research that had a general objective to evaluate the implementation of CS control interventions in prenatal care in PHC units. This research project was approved by the Research Ethics Committee of the *Universidade Federal de Juiz de Fora* (UFJF/MG) (CAAE: 80417117.2.0000.5147; # 3.749.587). All participants signed an informed consent form.

## RESULTS

A total of 416 postpartum women were evaluated in this study. Seventeen women were not interviewed because they met the exclusion criteria or refused to participate in the research, totaling 399 women (36 with GS) ( Figure 1 ). The prevalence of GS was 9.61% (95%CI: 7.14-12.83). Tables 1 and 2 show the distribution of the variables of interest in the study population, the prevalence of syphilis, and its associated factors.

**Table 1:** Socioeconomic, demographic, and behavioral characteristics of women who underwent prenatal care at a primary healthcare unit, according to syphilis diagnosis

Variables	Syphilis ^†^ diagnosis				

	n (%)*	No n (%)	Yes n (%)	PR (95%CI)	p value
Block I - Socioeconomic and demographic characteristics					
Age group					
≥25 years	185 (46.4)	172 (47.4)	13 (36.1)	1	
15 to 24 years	214 (53.6)	191 (52.6)	23 (63.9)	1.52 (0.79-2.93)	0.201
Skin color					
White/yellow	121 (30.3)	107 (29.5)	14 (38.9)	1	
Brown/black	278 (69.7)	256 (70.5)	22 (61.1)	0.68 (0.36-1.29)	0.242
Marital status					
Living with partner	296 (74.0)	275 (75.7)	21 (58.3)	1	
Does not live with the partner	56 (14.0)	52 (14.3)	4 (11.1)	1.00 (0.35-2.82)	0.990
Does not have a partner	47 (12.0)	36 (10.0)	11 (30.6)	3.29 (1.70-6.39)	<0.001
Education level					
Completed high school	143 (35.9)	136 (37.5)	7 (19.4)	1	
Completed elementary II	176 (44.1)	158 (43.5)	18 (50.0)	2.08 (0.89-4.86)	0.088
Completed elementary l	80 (20.0)	69 (19.0)	11 (30.6)	2.80 (1.13-6.96)	0.026
Occupational status					
Paid activity	170 (42.6)	156 (43.0)	14 (33.3)	1	
Unpaid	229 (57.4)	207 (57.0)	22 (66.7)	1.16 (0.61-2.21)	0.638
Per capita income (MW)*					
≥0.5	167 (44.1)	160 (46.7)	7 (19.4)	1	
<0.5	212 (55.9)	183 (53.3)	29 (80.6)	3.26 (1.46-7.27)	0.004
Block II - Behavioral characteristics					
Age of first sexual intercourse*					
≥16 years	217 (54.8)	205 (57.0)	12 (33.3)	1	
≤15 years	180 (45.2)	155 (43.0)	24 (66.7)	2.41 (1.23-4.68)	0.009
Use of condoms during pregnancy ^#^					
At all times	15 (4.0)	14 (4.0)	1 (3.1)	1	
Mostly	19 (5.0)	18 (5.2)	1 (3.1)	0.78 (0.05-1.64)	0.863
Less than half the time	43 (11.4)	33 (9.6)	10 (31.3)	3.48 (0.48-25.07)	0.214
Not once	301 (79.6)	281 (81.2)	20 (62.5)	0.99 (0.14-6.95)	0.997
Number of sexual partners during pregnancy					
One	387 (96.7)	354 (97.5)	33 (91.7)	1	
Two or more	12 (3.3)	9 (2.5)	3 (8.3)	2.93 (1.04-8.24)	0.041
History of syphilis (pregnant woman)					
No	388 (97.3)	360 (99.2)	28 (77.8)	1	
Yes	11 (2.7)	3 (0.8)	8 (22.2)	10.07 (6.05-16.76)	<0.001
History of syphilis (partner)					
No	376 (94.9)	354 (97.8)	22 (64.7)	1	
Yes	20 (5.1)	8 (2.2)	12 (35.3)	10.25 (5.96-17.62)	<0.001
History of STI					
No	335 (83.9)	313 (86.2)	22 (61.1)	1	
Yes	64 (16.1)	50 (13.8)	14 (38.9)	3.33 (1.80-6.16)	<0.001
Alcohol consumption during pregnancy					
No	309 (77.4)	286 (78.8)	23 (63.9)	1	
Yes	90 (22.6)	77 (21.2)	13 (36.1)	1.94 (1.02-3.67)	0.042
Illicit drug use (pregnant woman)					
No	370 (92.7)	339 (93.4)	31 (86.1)	1	
Marijuana and others	19 (4.8)	15 (4.1)	4 (11.1)	2.51 (0.98-6.39)	0.053
Crack and cocaine	10 (2.5)	9 (2.5)	1 (2.8)	1.19 (0.17-7.91)	0.855
Illicit drug use (partner)*					
No	310 (79.9)	296 (83.9)	14 (40.0)	1	
Marijuana and others	48 (12.4)	39 (11.0)	9 (25.7)	4.15 (1.90-9.06)	<0.001
Crack and cocaine	30 (7.7)	18 (5.1)	12 (34.3)	8.85 (4.5-17.39)	<0.001

n=399 postpartum women; ^†^ Syphilis: determined by treponemal and/or non-treponemal tests; * differences are justified by the options “don’t know,” “don’t remember,” and “no record”; ^#^ 20 women reported not having had sex during pregnancy.

PR: prevalence ratio; 95%CI: 95% confidence interval; MW: minimum wage; STI: sexually transmitted infection.

**Table 2:** Reproductive characteristics and access and use of health services by women who received prenatal care at a primary healthcare unit, according to syphilis diagnosis

Variables	n (%)*	Syphilis ^†^ diagnosis			

		No n (%)	Yes n (%)	PR (95%CI)	p value
Block III - Reproductive characteristics					
Age at first pregnancy					
≥25 years	64 (16)	60 (16.5)	4 (11.1)	1	
16 to 24 years	274 (68.7)	252 (69.5)	22 (61.1)	1.28 (0.45-3.60)	0.634
≤15 years	61 (15.3)	51 (14.0)	10 (27.8)	2.62 (0.86-7.93)	0.088
Number of pregnancies					
≤3 pregnancies	328 (82.2)	300 (82.6)	28 (77.8)	1	
≥4 pregnancies	71 (17.8)	63 (17.4)	8 (22.2)	1.31 (0.62-2.77)	0.464
History of miscarriage					
No	333 (83.5)	305 (84.0)	28 (77.8)	1	
Yes	66 (16.5)	58 (16.0)	8 (22.2)	1.44 (0.68-3.02)	0.333
Stillbirth history					
No	395 (99.0)	361 (99.4)	34 (94.4)	1	
Yes	4 (1.0)	2 (0.6)	2 (5.6)	5.80 (2.06-16.31)	0.001
Low weight history					
No	360 (90.2)	325 (89.5)	35 (97.2)	1	
Yes	39 (9.8)	38 (10.5)	1 (2.8)	0.26 (0.37-1.87)	0.183
History of prematurity*					
No	356 (89.7)	323 (89.5)	33 (91.7)	1	
Yes	41 (10.3)	38 (10.5)	3 (8.3)	0.78 (0.25-2.46)	0.684
Block IV - Access and use of health services in prenatal care					
Prenatal PHC model					
With FHS	282 (70.7)	251 (95.4)	31 (86.1)	1	
Other	117 (29.3)	112 (4.6)	5 (13.9)	0.38 (0.15-0.97)	0.044
Number of prenatal consultations					
≥6 consultations	301 (75.4)	279 (76.9)	22 (61.1)	1	
≤5 consultations	98 (24.6)	84 (23.1)	14 (38.9)	1.95 (1.04-3.67)	0.037
Start of prenatal care					
First trimester	238 (59.7)	221 (60.9)	17 (47.2)	1	
Second trimester	141 (35.3)	126 (34.7)	15 (41.7)	1.48 (0.76- 2.89)	0.239
Third trimester	20 (5.0)	16 (4.4)	4 (11.1)	2.80 (1.04-7.53)	0.042
Syphilis test					
None	9 (2.3)	8 (2.2)	1 (2.8)	1	
One	108 (27.0)	101 (93.5)	7 (19.4)	0.58 (0.80-4.24)	0.594
Two or more	282 (70.7)	254 (70.0)	28 (77.8)	0.89 (0.13-6.87)	0.907
Gestational age at first syphilis test					
First trimester	181 (45.4)	168 (46.3)	13 (36.1)	1	
Second trimester	168 (42.1)	149 (41.0)	19 (52.8)	1.57 (0.80-3.09)	0.681
Third trimester	39 (9.8)	36 (10.0)	3 (8.3)	1.07 (0.31-3.58)	0.187
During childbirth	11 (2.7)	10 (2.7)	1 (2.8)	1.26 (0.18-8.83)	0.812
HIV tests					
None	11 (2.5)	9 (2.5)	2 (5.6)	1	
One	149 (37.6)	137 (37.7)	12 (33.3)	0.44 (0.11-1.73)	0.245
Two or more	239 (59.9)	217 (59.8)	22 (61.1)	0.50 (0.35-1.88)	0.311
Gestational age at first HIV test ^*^					
First trimester	183 (46.2)	170 (47.2)	13 (36.0)	1	
Second trimester	165 (41.8)	146 (40.6)	19 (52.8)	1.62 (0.82-3.18)	0.160
Third trimester	40 (10.0)	38 (10.5)	2 (5.6)	0.70 (0.16-3.00)	0.635
During childbirth	8 (2.0)	6 (1.7)	2 (5.6)	3.51 (0.94-13.05)	0.060

n=399 postpartum women. ^†^ Syphilis: determined by treponemal and/or non-treponemal tests; * differences are justified by the options “don’t know,” “don’t remember,” and “no record.”

PR: prevalence ratio; 95%CI: 95% confidence interval; PHC: primary healthcare; FHS: Family Health Strategy.

Their ages ranged from 15 to 47 years (mean± standard deviation [SD]: 25±5.8), 69.7% self-reported brown or black skin color, 12.0% reported not having a partner, and 64.1% had completed elementary school or less. Approximately 60.0% of the women did not have a paying job and had a low income.

Approximately half of the women evaluated (45.2%) reported that their first sexual intercourse was before 15 years of age (11-36, mean 16.0±2.5). Most women (344/91.0%) reported having sexual intercourse during pregnancy (12 with more than one partner) without using a condom or using one less than half of the time.

These findings demonstrate that a large proportion of these women are at risk of being infected with STIs. Eleven (2.7%) women reported having had syphilis and 20 (5.1%) reported that their partners had had syphilis prior to their current pregnancy. Sixty-four (16.1%) women reported previous STI signs and symptoms.

Women reported consumption of alcohol (22.6%) and illicit drug use (7.3%) by themselves, and illicit drug use by their partners (20.1%). The drugs reported included cocaine, crack, “freebase,” sniffs drugs (“ *lança perfume* ”), and marijuana.

The age at first pregnancy ranged from 11 to 46 years (mean, 19.9±4.9). The number of pregnancies was three or less for 82.2% (range, 1-11; mean, 2.3±1.5). The women also reported a history of abortion (16.5%), stillbirth (1.0%), perinatal mortality (1.3%), low birth weight (9.8%), and premature birth (10.3%).

Regarding prenatal care, 70.0% of the women received prenatal care from FHS teams, 59.7% started prenatal care in the first trimester, and 75.4% had six or more consultations. Many of the women received at least one test for syphilis (97.7%) and some received two or more (70.7%). They also received at least one test for HIV (97.5%) and some received two or more tests (59.9%). Less than half of the women underwent their first tests during the first trimester.

Variables with p≤0.20 in the bivariate analysis (Tables 1 and 2) were included in the regression models. Not having a partner, low income, and low education were independently associated with GS; however, in the multivariate analysis of this block, low education was not significant. The prevalence of GS among women who had no partner and had low income was approximately three times the prevalence among those without these factors.

Having two or more partners during pregnancy, history of syphilis, alcohol consumption during pregnancy, history of other STIs, first sexual intercourse by the age of 15, partners with a history of syphilis, and partners that were users of crack/cocaine or other illicit drugs were independently associated with GS. In the multivariate analysis of this block, the associations between the last five factors were retained. The prevalence of GS among women with partners who were crack/cocaine users or who had a history of syphilis was approximately six times higher than among those without this history. The prevalence of GS among those whose partners were marijuana/other illicit drug users was approximately three times higher than among those who did not. The prevalence of GS among women who had their first sexual intercourse by the age of 15 a history of STIs was twice that of women without these factors.

A history of stillbirth was independently associated with GS and remained significantly associated (five times the prevalence compared with those with no history of stillbirth) in the multivariate analysis of the reproductive traits block.

Having received prenatal care from FHS teams, attending five appointments or less, and starting care in the third trimester were independently associated with GS. However, in the multivariate analysis of this block, the number of appointments lost significance. The prevalence of GS among women who started prenatal care in the third trimester or received prenatal care from FHS teams was approximately three times higher than those without this history.

Table 3 presents the multivariate analysis of each block and the Poisson regression models. Table 4 shows the results of the multivariate analysis after adjustment between the blocks and Poisson regression models. The variables that represent the behavioral characteristics and history of STIs absorbed the effects of the other variables, leaving only those in the final model. The prevalence of GS among women with partners who are crack/cocaine users or with a history of syphilis was approximately six times the prevalence among women without this history. The prevalence of GS in women whose partners were marijuana/other illicit drug users was approximately three times higher than in women without this history. The prevalence of GS in women with a history of STIs or first sexual intercourse before 15 years of age was twice that in women without this history.

**Table 3:** Poisson regression model by blocks, with crude and adjusted prevalence ratios, 95% confidence intervals, and p values, among the selected variables and syphilis diagnosis in women who underwent prenatal care at a primary healthcare unit

Variables	Syphilis ^†^ diagnosis (yes)					

	Crude PR	95%CI	p value	Adjusted PR	95%CI	p value
Block I - Socioeconomic and demographic characteristics						
Marital status						
Living with partner	1	-		1	-	
Does not live with partner	1.006	0.35-2.82	0.990	0.94	0.34-2.56	0.904
Does not have a partner	3.29	1.70-6.39	<0.001	3.07	1.44-5.28	<0.002
Per capita income						
≥0.5	1	-		1	-	
<0.5	3.26	1.46-7.27	0.004	2.85	1.28-6.3	0.010
Block II - Behavioral characteristics						
Age of first sexual intercourse						
≥16 years	1	-		1	-	
≤15 years	2.41	1.23-4.68	0.009	2.42	1.24-4.71	0.009
History of STI						
No	1	-		1	-	
Yes	3.33	1.80-6.16	<0.001	2.30	1.25-4.22	0.007
History of syphilis (partner)						
No		-		1	-	
Yes	10.25	5.96-17.62	<0.001	5.98	2.97-12.06	<0.001
Illicit drug use (partner)						
No	1					
Marijuana and others	4.15	1.90-9.06	<0.001	3.02	1.29-7.04	0.011
Crack and cocaine	8.85	4.5-17.39	<0.001	6.42	3.14-13.15	<0.001
Block III - Reproductive characteristics						
Stillbirth history						
No	1	-		1	-	
Yes	5.80	2.06-16.31	<0.001	5.21	1.48-18.32	<0.010
Block IV - Access and use of health services in prenatal care						
Prenatal PHC model						
With FHS	1	-		1	-	
Without FHS	0.38	0.15-0.97	0.028	0.35	0.13-0.92	0.033
Start of prenatal care						
First trimester	1	-		1	-	
Second trimester	1.48	0.76-2.89	0.239	1.60	0.82-3.12	0.163
Third trimester	2.80	1.04-7.53	0.042	3.15	1.21-8.22	0.019

^†^ Syphilis: determined by treponemal and/or non-treponemal tests.

STI: Sexually transmitted infection; PR: prevalence ratio; 95CI%: 95% confidence interval; PHC: primary healthcare; FHS: Family Health Strategy.

**Table 4:** Final model of Poisson regression, with crude and adjusted prevalence ratios, 95% confidence intervals, and p values between the selected variables and syphilis diagnosis in women who received prenatal care at a primary healthcare unit

Variables	Syphilis ^†^ diagnosis (yes)					

	Crude PR	95%CI	p value	Adjusted PR	95%CI	p value
Block II - Behavioral characteristics						
Age of first sexual intercourse						
≥16 years	1	-		1		
≤15 years	2.41	1.23-4.68	0.009	2.42	1.24-4.71	0.009
History STI						
No	1	-		1	-	
Yes	3.33	1.80-6.16	<0.001	2.42	1.25-4.22	0.007
History of syphilis (partner)						
No		-		1	-	
Yes	10.25	5.96-17.62	<0.001	5.98	2.97-12.06	<0.001
Illicit drug use (partner)						
No	1			1		
Marijuana and others	3.02	1.90-9.06	<0.001	3.02	1.29-7.04	0.010
Crack and cocaine	6.85	4.5-17.39	<0.001	6.42	3.14-13.15	<0.001

^†^ Syphilis: determined by treponemal and/or non-treponemal tests.

PR: prevalence ratio; 95CI%: 95% confidence interval; STI: sexually transmitted infection.

## DISCUSSION

This study identified a high estimated prevalence of GS. Latin American and the Caribbean had the third highest estimated prevalence of GS in the world (0.42%), behind Africa (1.68%) and the Eastern Mediterranean (0.57%). ^( 1 , 6 )^ In Brazil, the estimated prevalence of GS ranges from 1.02% to 28.60%, depending on the source. ^( 8 , 15 - 18 )^ These variations can be partially explained by differences in the scope of the study, definition adopted for syphilis infection, data source, and inequalities in age distribution. ^( 8 , 17 )^

The variables that represent the behavioral characteristics and history of STIs were significantly associated with GS at all levels of analysis, highlighting the importance of these factors. It is possible that the increase in syphilis is explained by vulnerable situations that involve behavioral characteristics of unsafe sexual practices. ^( 10 , 16 , 19 )^ However, although they did not all remain in the final model, it is important to discuss the factors associated with GS in each block. Poverty results in vulnerable conditions, such as behavioral or reproductive, which may be associated with a decrease in access to and inadequacy of prenatal care. ^( 10 , 16 )^ We observed a higher prevalence of GS in women who started prenatal care later and had a lower frequency of appointments, demonstrating a close relationship between syphilis and prenatal care. ^( 8 , 10 , 15 , 16 )^

Early sexual initiation, as in other studies, may be related to a pattern of risky sexual behavior for STIs. ^( 10 , 16 )^ The fact that most women report never using condoms or using them in less than half of their sexual intercourse encounters during pregnancy reveals that a large proportion of women are at risk of contracting STIs. ^( 10 , 16 , 19 )^

In this study, some variables were investigated with only the women, and there was no interview with their sexual partners. Having a partner with a history of syphilis and/or illicit drug use was associated with the occurrence of syphilis in women. The incidence of CS can be reduced by using relatively simple interventions. However, as long as syphilis is prevalent among adults, the potential for mother-to-child transmission remains high. Thus, the sustainable elimination of CS requires coordinated efforts to simultaneously reduce the rate of acquired syphilis in adults. ^( 20 )^ In this sense, prenatal care provides an ideal opportunity to approach women about their sexual partnerships.

Contact tracing of the sexual partners of women with syphilis is an opportunity for the detection of syphilis in men, treatment, and reduction of the transmission chain. ^( 3 , 21 , 22 )^ However, it is known that contacting a partner is not easy if he is not participating in prenatal care. ^( 16 , 22 )^ We emphasize the need to implement the National Policy for Integral Attention to Men’s Health (PNAISH - *Política Nacional de Atenção Integral à Saúde dos Homens* ), which, through the partner’s prenatal care, points to an opportunity to connect this group to health units and carry out preventive actions beyond the control of syphilis. ^( 22 , 23 )^

The use of illicit drugs by partners is a vulnerability factor for GS, as found in other studies. ^( 10 , 18 , 24 )^ Risk behaviors for STIs associated with the use of illicit drugs include exchanging sex for drugs, not using condoms, a greater number of sexual partners, and unplanned sexual activity, among others. ^( 10 , 19 )^

Syphilis surveillance in the United States from 2013 to 2017 showed that the proportion of primary and secondary syphilis cases that reported injectable drug, methamphetamine, or heroin use (or that reported sex with a person who uses injectable drugs) in the past 12 months was more than double among women and among men who have sex with women only. ^( 24 )^

The remarkable increase in the number of primary and secondary syphilis cases among women of childbearing age is mirrored by the increasing number of CS cases and increasing infant mortality. ^( 4 , 24 )^ All stages of syphilis in pregnant women pose a risk of transmission to the fetus, but the risk is considerably higher in the early stages than in the later stages of syphilis. These data suggest a link between illicit drug use and the rise in CS in the United States. ^( 4 , 24 )^ Another study showed the temporal and individual relationship of the associations of three epidemics: the use of smoked cocaine (“freebase” and crack), STI associated with genital ulcers, and heterosexually transmitted HIV infection. ^( 25 )^

Similar to other studies, a greater number of sexual partners during pregnancy and history of syphilis or other STIs in the pregnant woman were both associated with syphilis in the current pregnancy. ^( 10 , 16 - 18 )^ The adoption of safe sexual behaviors does not depend solely on the education level, income, knowledge of safe sexual behaviors, and access to condoms, but also on the meanings attributed to sexuality and the care for one’s own health. ^( 10 )^ The actions proposed to mitigate syphilis among women still face the challenge of connecting prevention and care, and promoting sexual and reproductive autonomy. ^( 10 , 16 )^

Alcohol consumption during pregnancy is associated with GS, as has been reported in other studies. ^( 17 , 18 )^ Alcohol consumption increases the chance of risky behaviors, such as not using condoms and increasing the number of sexual partners. ^( 19 )^

Similar to other studies, low education and income were associated with GS. ^( 8 - 10 , 16 , 26 )^ The proportion of people affected by syphilis remains high among the poorest and most marginalized populations in the Americas, and Brazil has historically been among the most unequal countries in the region. ^( 1 )^ In Brazil, there is a direct association between education and income inequality in which salary differentials are associated with schooling. ^( 27 )^ Years of study can influence the way health problems are perceived, and interfere with the adequacy of prenatal care and syphilis treatment. ^( 27 )^

The association of syphilis with the report of a woman not having a partner has been found in other studies. ^( 26 )^ Socio-family insufficiency is one of the main health determinants of pregnant women. ^( 28 )^ Some authors suggest that having a partner is an enabling factor in the use of prenatal services. ^( 15 , 28 )^

Other studies have shown that women with GS have an increased risk of stillbirth. ^( 29 , 30 )^ Stillbirth history is important for suspected syphilis infections. ^( 3 , 29 , 30 )^

The association between receiving prenatal care from FHS teams and GS suggests problems in the functioning of these teams and/or can be attributed to the worse sociodemographic conditions of women residing in places where this strategy was primarily implemented in the municipalit chosen by the criterion of poverty ^( 9 , 31 )^ and mapped by the Institute of Applied Economic Research (IPEA) Hunger Map. ^( 31 )^ This map indicated that there were 17,000 families below the poverty line in Juiz de Fora. ^( 31 )^

As in other studies, our results indicate that women who start prenatal care later and have a lower frequency of appointments are at greater risk for the occurrence of GS and, consequently, its adverse effects. ^( 15 , 25 )^ Having performed “two or more tests for syphilis” was associated with both “beginning prenatal care in the first trimester” (79.8%, p<0.001) and having “six or more consultations” (89.0%, p<0.001) (data not shown in tables). This finding reveals that early initiation of prenatal care, continuity of care, and procedures for syphilis detection are an important part of the effectiveness of prenatal care. ^( 9 , 10 , 15 )^

An FHS team can minimize the effects of socioeconomic inequalities. ^( 9 , 12 )^ In some municipalities, in the basic network, it has already been demonstrated that pregnant women visited by community health workers start prenatal care earlier and have more prenatal appointments, laboratory tests, and counseling. ^( 9 )^ In addition, the implementation of rapid tests for syphilis in the PHC units, counseling, and immediate administration of penicillin would allow for the reduction of syphilis in the adult population, CS, and its more severe forms. ^( 1 , 32 )^

Figueiredo et al. carried out an ecological study using part of the data from the Brazilian Access and Quality Improvement Program in Primary Care (PMAQ-AB - *Programa de Melhoria do Acesso e da Qualidade na Atenção Básica)* assessment conducted in 2014. Their objective was to analyze the relationship between the approaches of diagnosis and treatment of syphilis in primary care and the incidence of GS and CS. Municipalities with reduced vertical transmission had higher median percentages of teams offering rapid tests (increased ability to detect GS) and patients taking penicillin, demonstrating a relationship between these actions and the reduction of CS. ^( 32 )^

These results indicate that syphilis is a health and social problem that is directly related to poverty, low education, and vulnerable lifestyles. ^( 1 , 5 , 7 - 10 , 16 )^ Vulnerable women should be offered specific prenatal care strategies that include sexual partnerships, structured actions for prevention, and assistance with active search, healthcare network support, and social services.

Limitations of this study include the possibility of gaps in information about tests for syphilis performed during prenatal care, especially for women without a diagnosis of syphilis, and omission or issuance of untrue information in responses regarding behavior. However, we reinforced the validity of the results by analyzing various data sources, correcting data collection instruments used in the pre-test and pilot study, and providing a welcoming environment at the time of the interview.

## CONCLUSION

These results reinforce that for the effective control of congenital syphilis, there is a need for specific interventions to control syphilis in the adult population. These interventions include expansion of access to quality prenatal care involving identification of the risks for syphilis and connection between prevention and treatment actions; implementation of strategies focused on early sexual education, which encourages the use of condoms from the beginning of sexual activity and guides the assessment of sexual and reproductive risks; establishment of prenatal care involving both partners; and effective implementation of the National Men’s Health Policy (PNAISH - *Política Nacional de Atenção Integral à Saúde dos Homens* ).
